# The Long and Winding Road to Uncertainty: The Link between Spatial Distance and Feelings of Uncertainty

**DOI:** 10.1371/journal.pone.0119108

**Published:** 2015-03-10

**Authors:** Tina Glaser, Joshua Lewandowski, Jessica Düsing

**Affiliations:** 1 Department of Psychology, University of Bielefeld, Bielefeld, Germany; 2 Joshua Lewandowski, School of Behavioral & Organizational Sciences, Claremont Graduate University, California, United States of America; 3 Jessica Düsing, Department of Psychology, University of Trier, Trier, Germany; University of Akron, UNITED STATES

## Abstract

Construal Level Theory (CLT) [1] defines psychological distance as any object, event, or person that cannot be experienced by the self in the here and now. The goal of the present research was to demonstrate that feelings of uncertainty are closely linked to the concept of psychological distance. Two experiments tested the assumption that spatial distance and uncertainty are bidirectionally related. In the first experiment, we show that perceived spatial distance leads to a feeling of uncertainty. The second experiment revealed that a feeling of uncertainty leads to a perception of greater distance. By demonstrating that distance is closely tied to uncertainty, the present research extends previous research on both distance and uncertainty by incorporating previously unexplained findings within CLT. Implications of these findings such as the role of uncertainty within CLT are discussed.

## Introduction

Life is a complex state of affairs that humanity constantly struggles to understand and predict. Unfortunately, life is filled with uncertainties that limit our ability to craft systematic predictions and models about the world around us. Looking at a cloudy sky right now might inform us that a storm is imminent—and to prepare accordingly—but those clouds provide little information regarding the weather a year from now. Similarly, knowledge about our immediate physical surroundings often fails to inform what occurs elsewhere. The further events occur in time or space the less information inherently exists about those events. However, the amount of information actually present about an event is sometimes different from the amount of information that is felt to exist. Thus, despite the amount of information being constant, individuals can feel more or less uncertain depending on how much information they feel is present. While it seems intuitive that more distant events are more uncertain than more proximal events due to differences in information, can distal events or objects to which the amount of information is finite or known still result in feelings of uncertainty? Specifically, do distant events and places indeed make us feel more uncertain, and do feelings of uncertainty also bring to mind more distant events and places?

In the present research, we tested whether distances in space are bidirectionally related to feelings of uncertainty. We propose that spatial distance is associated with increased feelings of uncertainty and vice versa. Uncertainty increases judgments of distance due to the perceived amount of information one has about a place. Previous studies have implicitly examined the relationship between distance and uncertainty [2, 3, 4], and the current investigation builds on this line of research by explicitly addressing whether distance and feelings of uncertainty are related.

## Theoretical Background

### Uncertainty

Uncertainty is often defined differently depending on the source of the uncertainty. For instance, causal uncertainty is lacking information about the cause of an event [5, 6]; personal uncertainty is a general unpredictability about one’s world [7]; attitude uncertainty is the validity of one’s convictions [8]; and task uncertainty is a lack of knowledge regarding how one will perform on a task [9]. In its most basic form, uncertainty is a state of not knowing [10, 11, 12].

Lacking knowledge can elicit one of at least two responses. One response is a ‘hot’, emotionally-driven reaction in which feelings of uncertainty are aversive, stressful, and anxious [9, 7]. They restrict our perspective and create an urge to alleviate this heightened response. The other response is a ‘cold’ cognitive state where missing information predisposes adopting a broader view that incorporates schematic information in order to make sense out of one’s limited knowledge [13, 14]. Uncertainty derived from missing information is a core component of the biases and heuristics research of the last 40 years [15, 16]. In this paper, we define uncertainty as a cognitive subjective feeling indicating to the individual that some information is missing. This feeling of uncertainty should elicit a broader, more distant mindset.

### Psychological Distance

The concept of psychological distance has become increasingly popular in recent years. This is not too surprising considering the growing evidence showing significant influences of psychological distance on a multitude of psychological variables such as judgments, attitudes, preferences, self-control, and information processing [17, 1]. Psychologically distant are those objects that cannot be experienced by the self in the here and now, and therefore have to be construed. The most prominent theory dealing with psychological distance is construal level theory (CLT) [18, 19, 1]. According to CLT, psychological distance is composed of four different kinds of distance: temporal, spatial, and social distance, as well as hypotheticality (i.e., less likely events are more distant and more likely events are less distant). One basic premise of CLT is that the mental representations of an event or object change as a function of the perceived distance to that object. In the far distance, objects and events are represented more abstractly (high level construals), whereas in the near distance, objects and events are represented more concretely (low level construals). This assumption of a bidirectional relation between distance and construal level is often described with the forest-tree analogy, stating that in the far distance you see the forest, whereas in the near distance you see the trees. Thus, in the near distance, our mental representations consist of concrete details, whereas in the far distance, only the big picture is mentally represented. These different levels of representation are explained with the fact that concrete information that goes beyond the abstract meaning of an object is either not available or not reliable in the far distance. Abstract representations, however, allow us to make better predictions about the future because they consist of invariant (i.e., temporally and spatially stable) features.

### Linking Uncertainty and Psychological Distance

There is an abundance of psychological research on both uncertainty and psychological distance that seem to co-exist without being linked to each other. Considering that it seems obvious that uncertainty and distance are related, it is even more surprising that there is—to the best of our knowledge—no study addressing this seemingly plausible relation and thereby linking these two lines of research. Feelings of uncertainty often result when information is unreliable, inaccessible, or unknowable. At the same time, perceiving distance often means that information seems unreliable, inaccessible, or unknowable [1]. Although CLT assumes that it is feelings of uncertainty about high-level vs. low-level features of an object or event that drives the psychological distance—construal level association [1], no study has directly tested whether this assumption holds true, despite key CLT findings being based on this assumption (discussed further below). Does distance indeed lead to a feeling of uncertainty? And can feelings of uncertainty lead to judgments of greater distance?

A number of studies related to CLT have shown that feelings of uncertainty and distance may be positively related. For instance, the view of uncertainty as a feeling of lacking knowledge is reflected in several studies that employ the Gestalt completion task. In this task, participants view a series of fragmented pictures of objects and attempt to recognize the “Gestalt” by mentally filling in the missing information. These studies provide support for the claim that when information seems incomplete, adopting a more distant mindset allows for the gaps to be filled in more effectively [2, 4, 20]. These findings, however, seem to be task-specific because studies using other tasks indicate that a near (rather than far) mindset can accomplish the task better [20]. Other studies show that adopting a distant perspective when completing a maze where obstacles are present allows for more effective navigation compared to adopting a more proximal mindset [3]. In this case, obstacles can be viewed as generating a sense of uncertainty in that participants are presented with a situation in which information about completing the maze seems unavailable or unreliable. In a similar vein, Nussbaum, Liberman, and Trope [21] examined the relationship between confidence in predicting near and distant events when given more or less knowledge of classic psychological experiments. They found a marginal effect that participants given less knowledge about psychological experiments are less confident about predicting distant results, suggesting that by increasing feelings of uncertainty, more distant events seem to decrease confidence in predictions. Thus, previous research at least indirectly indicates that uncertainty and distance could be linked. However, these results are partly inconclusive which highlights the necessity of testing directly whether there is indeed a link between distance and uncertainty. Another series of experiments revealed that priming seemingly uncertain words such as “maybe” versus certain words like “sure” in scenarios congruent in terms of distance (i.e., “maybe”/distant and “sure”/proximal) resulted in quicker reaction times in a Stroop-like task, where participants had to classify either words or spatial distances [22], suggesting that feelings of uncertainty and distance are interrelated when compared to certainty and proximity. Furthermore, the studies of Bar-Anan and colleagues [22] consistently revealed a distance main effect, meaning that participants needed longer to react to the distant stimuli as compared to the proximal stimuli. However, this was not the focus of their study and the authors even argue that CLT has no prediction about the observed main effects in these studies. They hypothesize that “it might result from the fact that the proximal targets were bigger in size, or maybe proximal targets are more important than distal targets” ([22], p. 614). Bar-Anan and colleagues argue further that future research is needed to explore the real causes of this effect. The present research explicitly builds on this finding. Specifically, we argue that feeling uncertain is a plausible cause underlying the longer reaction times associated with psychological distance.

It is important to acknowledge that a large body of research has examined the relationship between probability estimates (“hypotheticality”) and psychological distance and found that higher psychological distance is associated with lower probability estimates [23, 24, 20]. The current research differs from these efforts in that we examine perceived uncertainty rather than discrete probability estimates. Although subjective uncertainty and discrete probabilities are similar, these two constructs are clearly not identical. Thus, a distinction between numeric and verbal expressions of uncertainty is important because probability estimates do not always map onto subjective uncertainty [25, 26, 27, 28]. For instance, Windschitl and Weber [28] asked participants to imagine going on a holiday trip either in Hawaii or India and that, given their blood type, they had a 20% chance of contracting Malaria. Despite being provided the exact same probability estimate, reported likelihood estimates differed by location. In fact, this study provides evidence inconsistent with CLT because participants reported a higher, not lower, likelihood of an event occurring (contracting Malaria) in a more distant location (India) than in a closer location (Hawaii). So while the probability-as-distance framework supports the positive relationship between distance and construal, no such research exists when probabilities are replaced with the feeling of uncertainty—or the lack of a probability estimate.

By examining subjective uncertainty—rather than discrete probabilities—the current study provides a link to a host of seemingly unrelated social psychological phenomena and CLT. For instance, Social Identity Theory and the Identity-Uncertainty Hypothesis [9] are two commonly explored topics that hinge on the induction of perceived uncertainty with no mention of probability. Therefore, the current research provides a novel test of CLT by considering *subjective* uncertainty as an important variable associated with distance.

### Overview of Experiments

Previous studies provide indirect and preliminary support for the hypothesis that feelings of uncertainty are linked to psychological distance. With the present research, we aim to demonstrate explicitly that distance leads to feeling uncertain and vice versa. In Experiment 1, we intend to replicate the distance main effect obtained by Bar-Anan et al. [22] using a different paradigm that enables us to directly test whether this distance main effect is due to higher perceived uncertainty in the distance. First, we employ a unique operationalization of uncertainty within the CLT literature via signal detection. Rather than asking participants directly about their subjective uncertainty, we measure their hesitation in making a judgment, which is an unobtrusive method for assessing feelings of uncertainty [29, 30, 31, 32, 33]. A signal detection task is applied in which signals were presented on top of landscape pictures implying depth. The signals were presented in either a proximal or distant position from the observer. Second, we also varied the strength of the signal (strong signal vs. weak signal vs. noise). Participants’ task was to decide, via key press, whether a signal is present or not. Thus, signal strength was varied mainly in order to make the task more plausible because categorizing a signal makes sense only when the signal strength varies from trial to trial. Interestingly, there are two possibilities how signal strength could moderate the effect. On the one hand, the expected difference in reaction times for near vs. far signals may be most pronounced for weak signals as compared to strong signals and noise. The weak signal is ambiguous and it is difficult to detect whether the signal is present or absent, thus eliciting uncertainty (i.e., a subjective feeling of not knowing whether there is a signal or not) additionally to the uncertainty elicited by distance. This “double uncertainty” might add up and therefore lead to a more pronounced difference in reaction times compared to the strong signal and the noise presented in the far vs. near distance. On the other hand, recent results by Maglio, Trope, and Liberman [34] suggest a reverse influence such that the difference in reaction times is less pronounced for the weak signal. Their results indicate a sub-additive property of distance dimensions such that an initial priming of distance decreases the influence of subsequent distances. Applied to our study, it could be that for more uncertain tasks (i.e., when signal strength is weak), subsequent responses will be less sensitive to the distance condition. In this case, sensitivity to the uncertainty caused by distance might be reduced and as a consequence, the difference in reaction times for the near and far signals might be attenuated. Although this hypothesis is not the focus of the study, it provides us an interesting opportunity to test a facet of CLT. Taken together, we hypothesize that participants would take longer (i.e., would be more uncertain) to react to signals in the far vs. near distance. However, this effect might be qualified by task uncertainty (i.e., signal strength), whereby both directions of influence seem possible and are subject to investigation.

Experiment 2 tested whether a heightened feeling of uncertainty also leads to perceptions of greater distance. In this experiment, participants were primed with either feelings of certainty or uncertainty by reading sentences that were related either to certainty or uncertainty. Subsequently, they were presented with various pictures implying depth and had to estimate the distance they would have to walk from the bottom of each picture to an arrow that was pointing to a location on the picture. Our hypothesis was that participants give higher distance estimations after reading uncertain as compared to certain sentences.

### Ethics Statement

In both studies, all participants provided oral informed consent. We did not obtain written informed consent in order to protect participants’ anonymity. The experimenter documented consent by making a note in the research protocol. This consent procedure as well as the procedure of both experiments was approved by the Ethics Committee of the University of Bielefeld. Labeled datasets from both studies may be obtained by writing to the first author.

## Experiment 1: Causal Link from Distance to Uncertainty

The goal of Experiment 1 was to investigate whether psychological distance leads to a feeling of uncertainty. To test this, a signal detection task was applied. Participants were presented with landscape pictures implying depth. Psychological distance was varied by presenting signals (i.e., squares) on top of these pictures in either a proximal or distal position from the observer. Additionally, the strength of the signal was varied, such that strong signals, weak signals, or only noise was presented. Participants’ task was to decide as quickly as possible whether a signal was presented or whether there was only noise. Uncertainty was measured by assessing participants’ reaction times. We hypothesized that participants would display longer reaction times (i.e., show higher uncertainty) when reacting to the signals displayed in the distant vs. proximal position. Furthermore, we explored whether this effect is moderated by task uncertainty (i.e., signal strength).

### Participants and Design

Sixty-one students (16 male, 45 female) participated in exchange for course credit. The experiment consisted of a 2 (distance: near vs. far) × 3 (signal strength: noise only vs. weak vs. strong) within-subjects design.

### Materials and Measures

**Fig 1:**
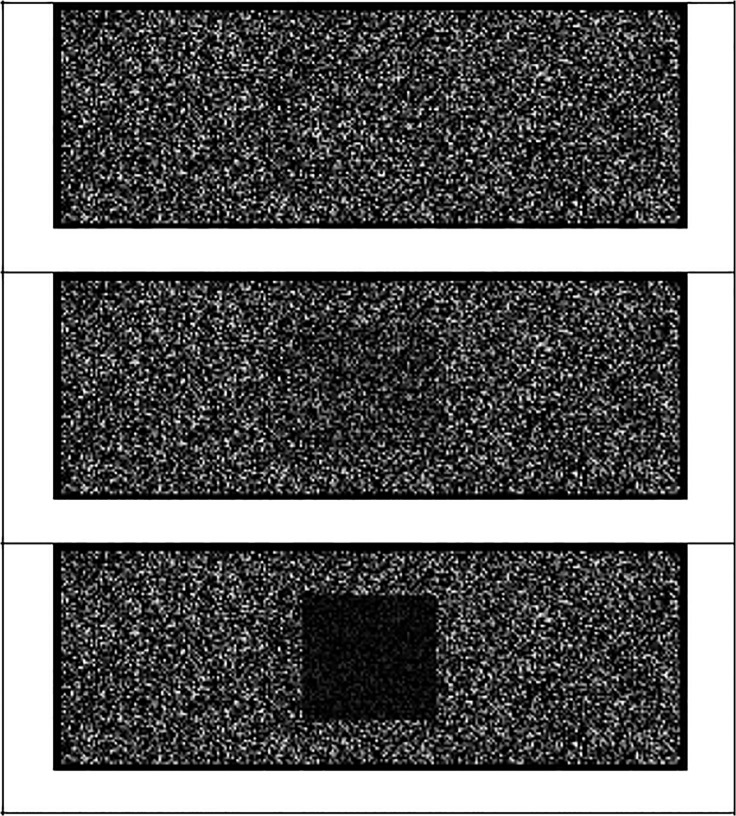
The three signals used in Experiment 1. The uppermost picture is noise, the middle picture is the weak signal and the lowermost picture is the strong signal.

We used 15 landscape pictures implying depth that had also been used in studies by Hansen and Wänke [35]. The stimuli that were used as signals were squares that were presented within a pixel array. The strength of the signals was varied by varying the amount of pixeling of the squares. In a pretest, it was determined which degree of pixeling of the square is very easy to detect (strong signal) and which degree of pixeling is quite difficult to detect (weak signal). In the pretest (N = 8), we presented 53 squares within a pixel array that were all pixeled to a different degree. The stimuli were presented on a white screen with no background picture. Each of the squares was presented once in the upper half of the screen and once in the lower part of the screen, resulting in 106 trials. Participants’ task was to decide via key press whether a signal (i.e., square) was present or not. Our criterion for the selection of the strong signal was that all pretest participants were able to detect the square within the pixels. This criterion was reached at 80% of pixeling. The criterion for the weak signal was that only half of the participants of the pretest detect the signal, whereas the other half does not detect the signal. This criterion was reached at 24% of pixeling. Thus, whether the weak signal is categorized as present or absent is on chance level, thereby ensuring that categorization of the weak signal is more difficult and ambiguous than categorization of the strong signal or the noise. The noise-only stimuli contained no square and participants could only see the pixel array (see Fig. 1). Additionally, we varied the position of the signal (i.e., upper vs. lower part of the screen) in order to rule out that participants generally need longer when a signal is presented in the upper vs. lower part of the screen. If this were the case, longer reaction times for stimuli that are presented on background pictures and are perceived to be distal from the observer could be due to the mere position of the stimulus on the screen, which would have nothing to do with a perception of higher distance. However, no differences in reaction times to the upper vs. lower position on the screen could be observed, thus ruling out that the confound of mere position on the screen and perceived distance can account for reaction time differences expected in Experiment 1.

**Fig 2:**
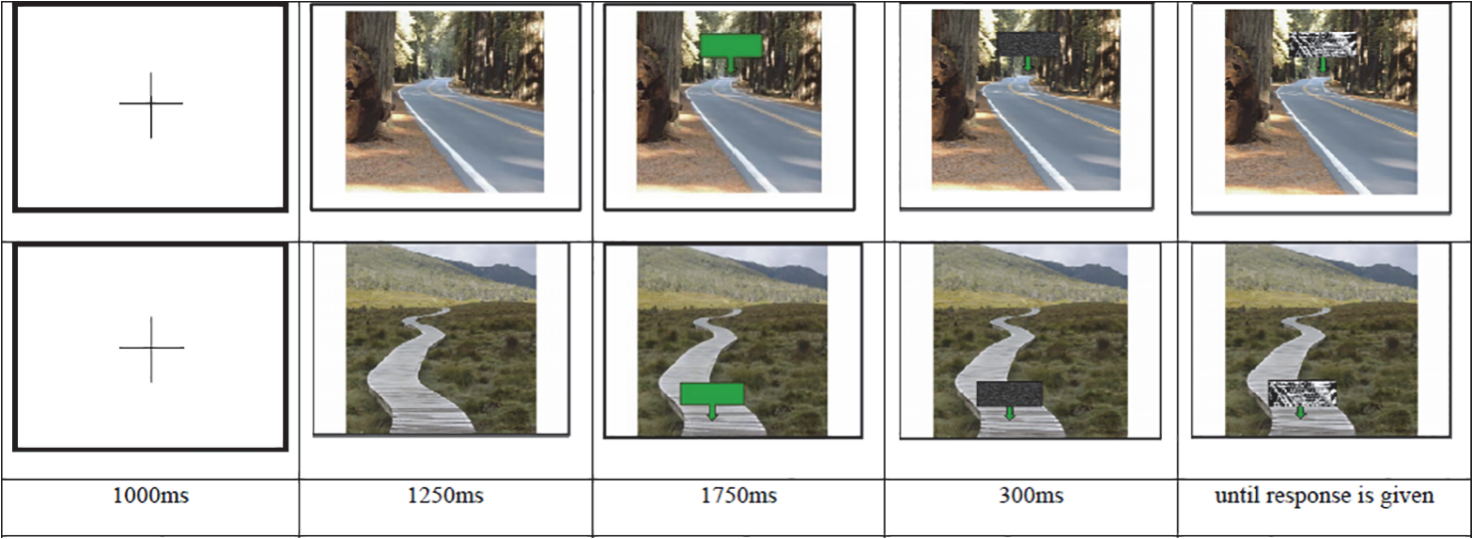
Temporal sequence of the presentations in the signal detection task in Experiment 1. In the first row, a weak signal is presented in a distal location. In the second row, a strong signal is displayed in a proximal location. In the third row, the presentation times for each picture are displayed.

In the signal detection task used in Experiment 1, the signals were presented on top of the 15 pictures. The position of the signals on the pictures varied. In half of the trials, the signals were presented in a position proximal to the participant, whereas in the other half of the trials the signals appeared in a position distal to the participant (see Fig. 2). Each trial began with a presentation of only the picture for 1250ms. Then an arrow appeared on the picture either in the near or far distance from the perspective of the participant. This arrow stayed on the screen for 1750 ms and served as a placeholder for the signal that then appeared within this arrow for 300ms. After 300ms, the signal was masked. As soon as the mask appeared, participants had to decide as quickly as possible whether they just saw a signal or not. Fig. 2 displays the temporal sequence of what participants saw. Each of the 15 pictures was presented four times with a signal in the near distance and four times with a signal in the far distance. This resulted in a total of 120 trials. The three different signal strengths were distributed equally over the two locations, such that each of the signal strengths appeared 20 times in the distal location and 20 times in the proximal location. The reaction times served as dependent variable.

### Procedure

When participants arrived at the lab, they were welcomed by an experimenter and seated in front of a computer screen. There were four computer workplaces in the room so that up to four people took part at the same time. Participants were told that we were interested in inter-individual differences in visual perception. The signal detection task was thoroughly explained to them. Specifically, participants were told that pictures of landscapes would be presented and that an arrow would appear on these pictures. Then, a “grey field” would appear within this arrow (i.e., the pixel array). Participants were informed that sometimes a square would be presented within this grey field. In some cases, it would be easy to detect the square and in some cases, it would be difficult to detect the square. Participants also received the information that the grey field would only appear for a very short time and would then be covered up by a black-and-white picture (i.e., the mask). They were told to press the key labeled with “yes” if they think that they saw a square and to press the key labeled with “no” if they did not see a square. It was emphasized that participants should react as fast and as accurately as possible. Participants first performed two practice trials in order to familiarize themselves with the task. They then completed the signal detection task. Finally, all participants were asked whether they had any guess regarding the hypothesis of the study. Demographics were also assessed.

### Results

Data from seven participants were dropped from the analyses because they were close in guessing the hypothesis of the study by mentioning that the study deals with uncertainty. However, the pattern of results was the same when including the hypothesis aware participants into the analyses. Reaction times that deviated more than three standard deviations from the mean reaction time of each condition (4.44%) were also excluded from the analyses.

**Fig 3:**
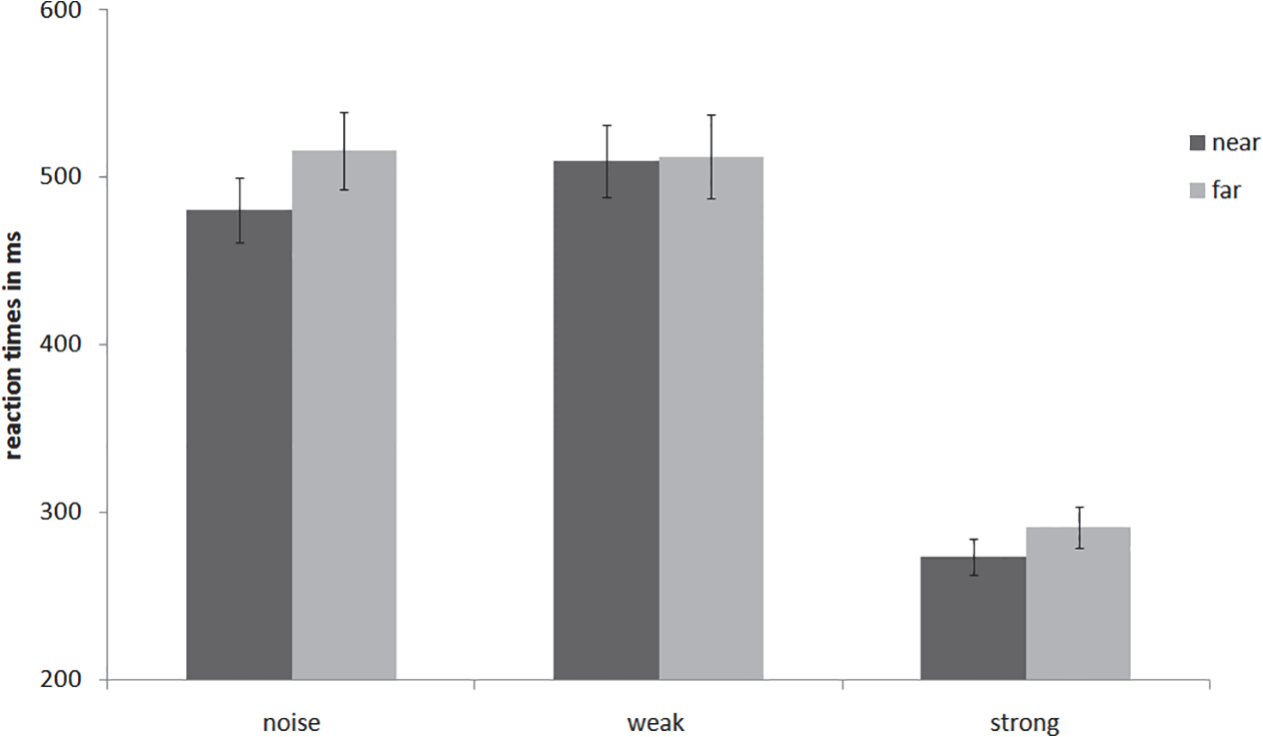
Reaction times in ms for the three signal strength in Experiment 1. Signals were either presented in the distal or proximal location. Error bars refer to standard errors.

An analysis of variance with repeated measures revealed a main effect of distance, *F*(1, 53) = 8.84, *p* = .004, η^2^ = .14, indicating that participants were indeed faster when the signal was presented in a near location (*M* = 423.63, *SD* = 118.79) vs. far location (*M* = 441.95, *SD* = 138.10). This finding corroborates Bar-Anan’s [22] finding and confirms our hypothesis that higher distance leads to feelings of uncertainty. Not surprisingly, there was also a main effect for signal strength, *F*(2, 106) = 95.33, *p* < .001, η^2^ = .64, showing that participants were faster when the signal was strong as compared to when the signal was weak or when there was only noise. The interaction effect between signal strength and distance was also significant, *F*(2, 106) = 3.76, *p* = .027, η^2^ = .07. Conducting separate planned comparisons for each signal strength revealed that there were significant differences in the predicted direction between near and far distance in the noise condition, *t*(53) = 3.75, *p* < .001, *d* = .22, and in the strong signal condition, *t*(53) = 3.15, *p* = .003, *d* = .20, but not in the weak signal condition, *t*(53) = .12, *p* = .91 (see Fig. 3). Thus, participants felt more uncertain and therefore needed longer to decide whether a signal was present or not when a strong signal or noise were displayed in the far as compared to the near distance. However, when the signal itself was weak, no difference in reaction time could be observed.

### Discussion

The results of Experiment 1 provide evidence that there is not only a link of uncertainty and distance but that distance causes a feeling of uncertainty. When having to decide whether a signal is present or not, participants felt more uncertain when this signal was displayed in a distal vs. proximal position. These findings provide a plausible explanation to previously unexplained CLT research, namely why longer reaction times are observed in the distance [22].

Furthermore, the strength of the signal moderated the reaction time difference. The difference in reaction times was only significant when either noise or strong signals were presented but not when the weak signal was presented, suggesting that the weak signal neutralizes the effects of distance. One possible explanation could be that the uncertainty caused by the weak signal overrides the uncertainty caused by distance. Thus, once uncertainty is already high (because the signal is weak), the effect of the subsequent, distance-related uncertainty is less. Support for this finding comes from recent research by Maglio and colleagues [34] who demonstrated that an initial instantiation of any psychological distance diminishes sensitivity to any other psychological distance. Similarly, an instantiation of uncertainty (i.e., deciding whether a signal is present) might reduce the sensitivity to an instantiation of another kind uncertainty (i.e., distance). However, it has to be taken into account that the research by Maglio and colleagues [34] explicitly referred to different types of distance and not to different kinds of uncertainty. Thus, whether such a sub-additive property of uncertainty dimensions can explain the obtained results remains speculative and future research should explore how different forms of uncertainty might interact. An alternative interpretation of the non-significant difference in reaction times for the weak signal in the near vs. far condition refers to the perceptual system per se. If the ability to detect a signal is very low in the first place (as it might be in the weak signal condition) the perceived distance might be irrelevant. This alternative interpretation is worth exploring in future research.

Expanding on the finding of Experiment 1 that distance leads to a feeling of uncertainty, we explore the bidirectionality of the effect in Experiment 2.

## Experiment 2: Causal Link from Uncertainty to Distance

The goal of Experiment 2 was to investigate whether feeling uncertain leads to a perception of higher distance. Participants had to silently read 24 individual sentences that were related either to certainty or uncertainty. Subsequently, they were presented with various pictures implying depth and had to estimate the distance they would have to walk from the bottom of each picture to an arrow which was pointing to a location on the picture. Our hypothesis was that uncertainty should lead to a perception of greater distance. Thus, participants should give higher distance estimations after reading uncertain as compared to certain sentences.

### Participants and Design

Sixty students (27 male, 33 female) participated in exchange for course credit. The experiment consisted of a one-factorial (priming: certainty vs. uncertainty) between-subjects design.

### Procedure

When participants arrived at the lab, they were welcomed by an experimenter and seated in front of a computer screen. Participants were told that they are about to take part in two short studies. In the first study, we were allegedly testing a new measure. Participants were instructed to imagine feeling like the person in the sentences. Twelve sentences that were either related to certainty (e.g., I feel certain, I am decided, the situation is clear, I know it) or to uncertainty (e.g., I feel uncertain, I am undecided, the situation is unclear, I don’t know it) were presented one after another on the screen and participants were asked to silently read each sentence. Each sentence remained on the screen for 15 seconds. The twelve sentences were presented twice in a fixed order. This induction of uncertainty is based on the mood induction procedure originally developed by Velten [36] and we pretested whether this priming procedure indeed elicited uncertainty. In a pilot study, N = 78 participants read the certain vs. uncertain sentences and subsequently answered a few questions about how they feel. Participants in the uncertain condition felt significantly more uncertain than in the certain condition (*t*(76) = 3.37, *p* = .001, *d* = .77). To avoid the potential bias of making participants aware that uncertainty was the effect of interest, this manipulation check was not included in the main study. In Experiment 2, after reading the sentences, participants were thanked for taking part in this first study and were introduced to the allegedly second study. They were told that it would be their task to estimate distances. To do so, several pictures implying depth that had already been used in Experiment 1 were presented on the screen. On each picture, a blue arrow was pointing to a location on the picture. The arrow was always placed randomly in a location that was neither very close nor very far from the bottom of the picture but always more or less in the middle. However, as the background pictures depicted different landscapes, the perceived distance from the bottom to the arrow varied depending on the respective picture. Importantly, though, the actual distance was not systematically varied but randomly chosen. Participants were asked to imagine standing at the bottom of the picture and walking to the marked location. They were asked to estimate how many meters they would have to walk from the bottom of the picture to the arrow. Participants were first shown an example picture. Subsequently, all 12 target pictures were presented one after another and participants had to type in their distance estimation for each picture. We also assessed participants’ mood (on a scale from 1 = bad mood to 10 = good mood) in order to rule out that mood rather than uncertainty drives the effects. Finally, demographics were assessed and participants were asked whether they had any guess regarding the hypothesis of the study.

### Results

Data of seven participants was removed from the analyses because they correctly guessed that there is a connection of the uncertainty manipulation and the distance estimations. However, the pattern of results was the same when including the hypothesis aware participants into the analyses. Before analyzing the distance estimations, values that deviated more than three standard deviations from the mean were excluded from the analyses (2.36%).

Confirming our hypothesis, a comparison of the distance estimations in both conditions revealed a significant difference, *t*(51) = 2.11, *p* = .039, *d* = .59. Participants in the uncertain condition provided significantly higher distance estimates (*M* = 17.99, *SD* = 10.53) than did participants in the certain condition (*M* = 12.79, *SD* = 7.29). Importantly, mood did not differ in the two conditions (*t* < 1), thus ruling out that mood is driving the effect. Furthermore, mood did not correlate with the distance estimations, *r* = .009, *p* = .95.

### Discussion

The results of Experiment 2 confirmed our hypothesis that uncertainty leads to greater perceived distance. Participants gave higher spatial distance estimations when they were primed with uncertainty as compared to when they were primed with certainty. Mood was unrelated to distance. Interestingly, this experiment shows that the relation between distance and uncertainty seems to be bidirectional such that greater distance leads to more uncertainty but that uncertainty also leads to greater estimates of distance.

## General Discussion

Although there is by now an abundance of research on the influence of distance on many different variables, the psychological meaning of distance is less clear. One essential question that arises from past research is what distance means psychologically. What could be one psychological construct underlying the phenomenon of psychological distance? The current investigation explored this question by building on CLT’s assumption that uncertainty is an important factor of psychological distance. Two experiments provide evidence that feelings of uncertainty are bidirectionally related to one dimension of psychological distance, namely spatial distance. Specifically, we found that distance causes increases in uncertainty (Experiment 1) and that uncertainty increases distance judgments (Experiment 2). These studies demonstrate that feelings of uncertainty and distance are interrelated and share a common meaning. Thus, experiencing distance also means experiencing feelings of uncertainty. Thinking of a far off place or a distant future also elicits feelings of uncertainty. Conversely, feeling uncertain also makes events or places seem further away and this relationship is not explained by mood.

How can this bidirectional relation of distance and uncertainty be explained? The finding that distance increases uncertainty seems quite intuitive. Experiencing distance also means experiencing some lack of information about certain aspects of the distant object, event, or person. Even when the amount of information objectively provided about a near or distant object does not differ, we subjectively experience that some information is missing in the distance. This subjectively perceived lack of knowledge is experienced as uncertainty [12]. Thus, the distance–uncertainty link may be comparable to the distance–construal link as it is postulated in CLT. Although the amount of information provided to participants in CLT studies does not differ, participants form high level construals of the distant event and low level construals of the concrete event. According to CLT, this is due to an association of distance and construal level, which became over-generalized because people have learned that it is functional to use high level construals when distance is high and low level construals when distance is low. We suggest that the distance–uncertainty relation may have developed for similar reasons. Thus, the subjective experience that information is missing in the distance could provide an explanation for the influence of distance on uncertainty.

Furthermore, this reasoning also seems to be able to explain the reverse influence from uncertainty to distance. We assume that it is deeply ingrained in humans that uncertainty is felt more in the distance than in proximity. Specifically, feeling uncertain about proximal objects, events, or persons is highly uncomfortable for us. For instance, we do not want to feel uncertain about our friends, our hometown, or tomorrow. However, feeling uncertain about a faraway place, an unknown person, or an event in the future seems much more tolerable and expected. Feelings of uncertainty should therefore bring to mind more distant rather than proximal aspects. Thus, we assume that by adopting a more distant perspective, the feeling of uncertainty can be alleviated or managed. This reasoning is in line with the notion that individuals manage and reduce uncertainty by using different strategies [37, 38, 39, 7]. Our results suggest that distancing oneself from people, places, or events seems to be one strategy of dealing with felt uncertainty. Recent CLT research supports this reasoning by demonstrating that participants feel less (causally) uncertain when an event is framed as distant vs. proximal [14].

After having demonstrated the existence of a bidirectional causal link between distance and uncertainty, it is interesting to note that recent research was able to also establish a link between uncertainty and construal level. Helzer and Edwards [13] found that causal uncertainty leads to more abstract construal of behavior which functions to restore structure and understanding. Thus, in terms of CLT, uncertainty not only leads to higher distance as demonstrated in the present research but also to higher levels of construal. Furthermore, Namkoong and Henderson [14] were able to demonstrate the opposite direction of causality. Specifically, they showed that abstract construals reduce causal uncertainty. However, the cited research only deals with causal uncertainty, leaving open whether the findings can be generalized to other types of uncertainty. Thus, in order to further understand how different types of uncertainty are related to distance and construal level in the framework of CLT, future studies should examine the relationship between distance, construal levels, and uncertainty. We hypothesize that uncertainty mediates the relation between distance and construal level. Distance leads to more uncertainty which in turn should lead to more abstract, high level information processing. Conversely, proximity should elicit certainty, which in turn should lead to the use of low level construals. Another line of future research should explore the differential effect of uncertainty about high-level versus low-level features of objects. If uncertainty increases psychological distance it should elicit a greater effect on low- versus high-level features of an object. Thus, psychological distance increases uncertainty about low-level features, which is why people form high-level construal of distant objects because high-level features are more stable, simplified, and certain relative to low-level features which are more variable, complex, and uncertain [40, 1].

## Concluding Remarks

Life is filled with varying uncertainties and our research is one of the first to explicitly demonstrate that distance is another cause for feeling uncertain and that a feeling of uncertainty is another reason for increased distance judgments. Although research on both distance and uncertainty has become increasingly popular in recent years, no studies had investigated whether these two important phenomena are directly related to each other. By bringing these two threads of research together and demonstrating that distance is closely tied to uncertainty, the present research extends previous research on both distance and uncertainty. Furthermore, our results enrich the knowledge about uncertainty by showing that uncertainty has an even more pervasive influence on social-cognitive processes than previously thought. By drawing connections between CLT and uncertainty, a more complete theoretical framework may be established for how decisions are made in everyday life: from how one prepares for an impending storm to making judgments about others.
